# Digital design of a hybrid bone and tooth-supported surgical guide in patients with unilateral few remaining natural teeth: a dental technique

**DOI:** 10.1186/s13104-024-06738-3

**Published:** 2024-03-18

**Authors:** Medhat Sameh Abdelaziz, Esraa M. Elshikh

**Affiliations:** 1 Department of Prosthodontics, Faculty of Oral and Dental Medicine, Future University in Egypt, Fifth Settlement, End of 90 Street, New Cairo, Cairo Egypt; 2 Ministry of Health, Cairo, Egypt

**Keywords:** Intra-oral scanning, Implants, Guided surgery, Hybrid surgical guides, Full arch implants

## Abstract

A technique for the design of a hybrid tooth and bone-supported implant drilling guide is described. The patient was scanned using cone beam computed tomography and an optical intraoral scanner. The dicom file was segmented according to the area of interest composed of bone and the remaining natural teeth. The Standard Tessellation Language (STL) file was trimmed to only the teeth providing support, followed by merging between the bone and teeth files in one STL. The implant drilling guide was designed with the Real Guide software program, and the file was 3-dimensionally printed in clear surgical guide resin. This technique offers an accurate, cost-effective digitally designed implant placement guide for patients with long-span distal extension edentulous areas and few remaining natural dentitions providing distal bone support. It can also be used in patients with hemi maxillectomy for zygomatic implant placement. This type of surgical guide provides more accuracy in implant surgeries that require flab elevation by gaining more support from the remaining natural dentition.

**Supplementary Information:**

The online version contains supplementary material available at 10.1186/s13104-024-06738-3.

## Introduction

Anatomic limitation such as maxillary sinus pneumatization or hemi maxillectomy makes the implant placement a challenging procedure and usually managed by sinus floor elevation, different types of regenerative techniques and bone grafting which requires extensive surgical procedures and time but these problems could be managed using guided zygomatic implants [1, 2].

In implant-guided surgeries, surgical guides are important tools used for transferring the digitally planned implant depth and angulations from the design software program to the patient's mouth [3–5]. Many factors influence the accuracy of guided surgeries, such as the number of remaining natural teeth that will provide surgical guide support and the quality of the guide design, starting from virtual data acquisition from the patient, and data alignment to guide manufacture by either 3D printing or milling [6, 7].

The type of structures providing support for the guide, such as teeth, bone, and mucosa, greatly affect the accuracy of guided surgery. Many previous reports have discussed the relationship between the type of guide support and the accuracy of implant placement in comparison to virtual planning, and they concluded that the more teeth supporting the guide with bilateral distribution, the greater the accuracy in transferring the implant position and angulation [8–10], On the contrary, many studies concluded that the bone-supported guides only showed the least accuracy [9–12].

Furthermore, the tooth-supported surgical guides used to place implant distal extension edentulous areas showed a large deviation in comparison to the virtual planning as these guides have only unilateral support, which may cause bending of the guide during implant drilling and placement due to a lack of appropriate support [8, 10].

The objective of this technique was to use digital technology to fabricate hybrid bone and teeth-supported implant placement guides that can be used in situations with few unilateral remaining natural teeth to provide double support from both bone and teeth.

## Technique

The technique starts with merging the bone and teeth data into one STL file, followed by implant planning and surgical guide design.

**Fig. 1:**
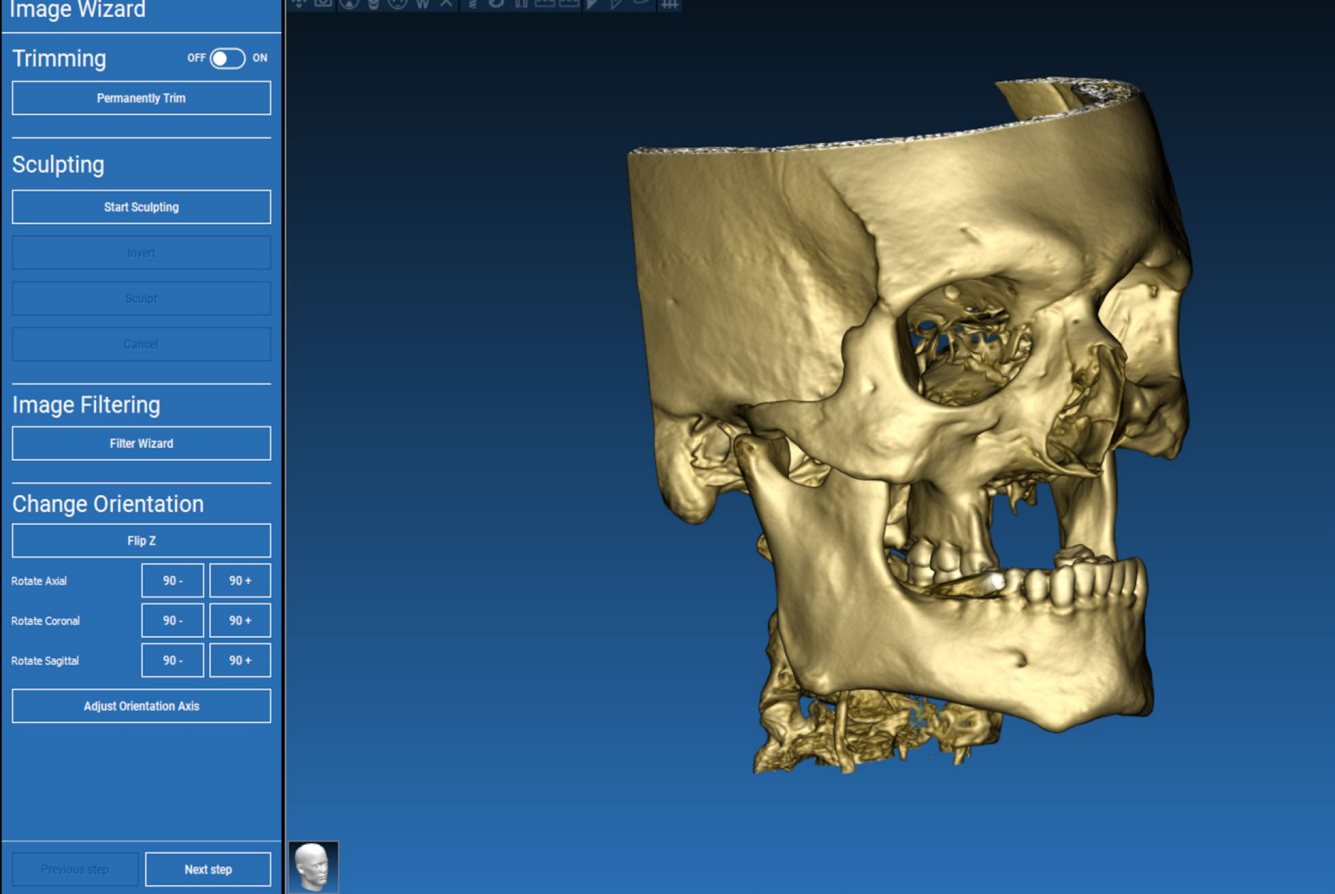
A Dicom file representing the bone and teeth of a patient with few remaining natural teeth and long-span distal extension edentulous area

**Fig. 2:**
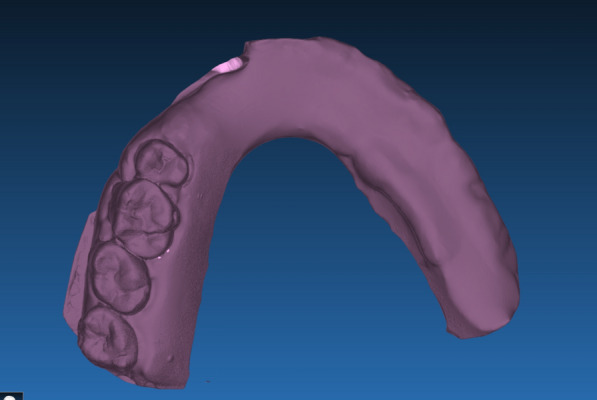
An STL file representing soft tissue and teeth of a patient with few remaining natural teeth and long-span distal extension edentulous area

**Fig. 3:**
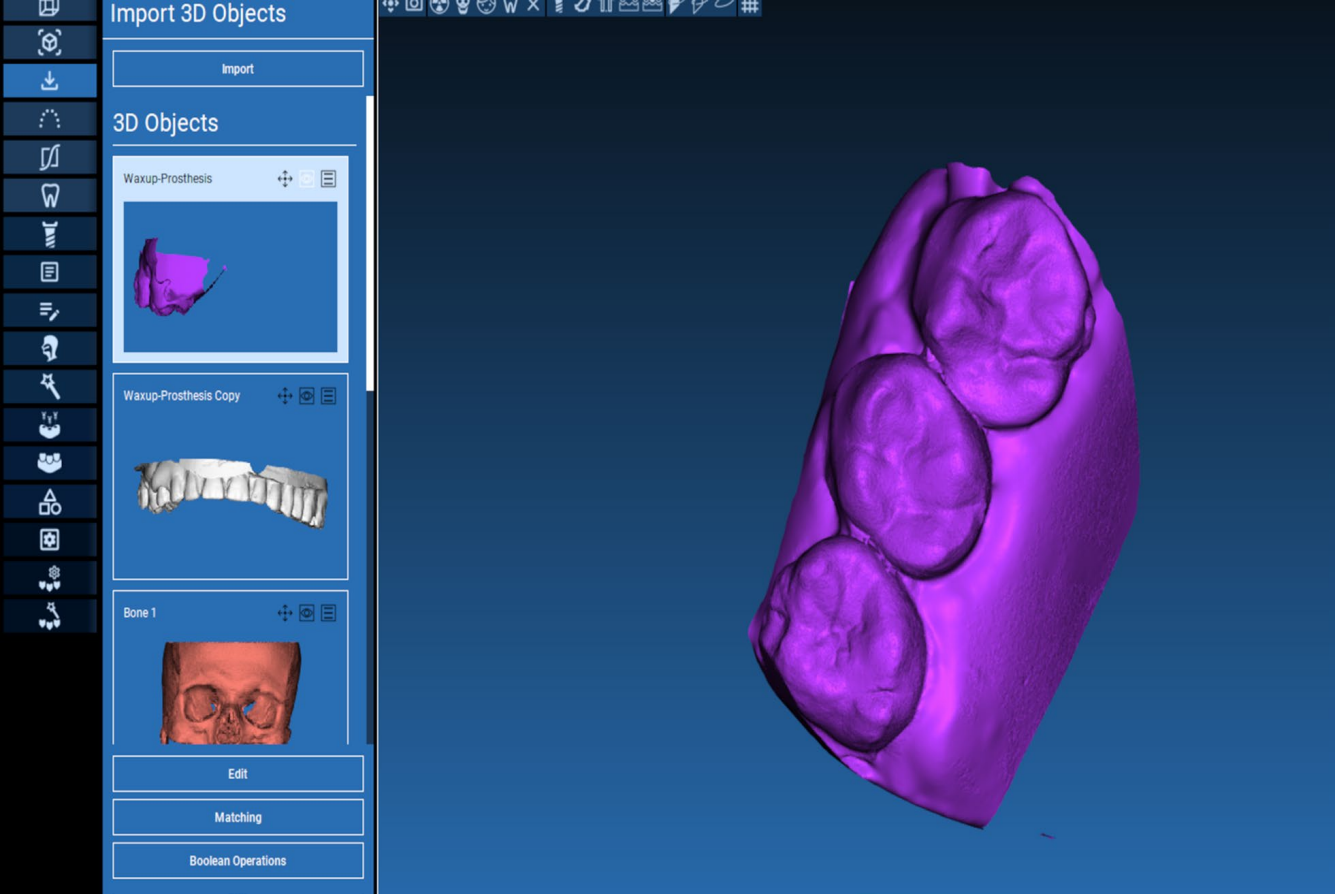
An STL file representing the teeth of interest and their soft tissue only

**Fig. 4:**
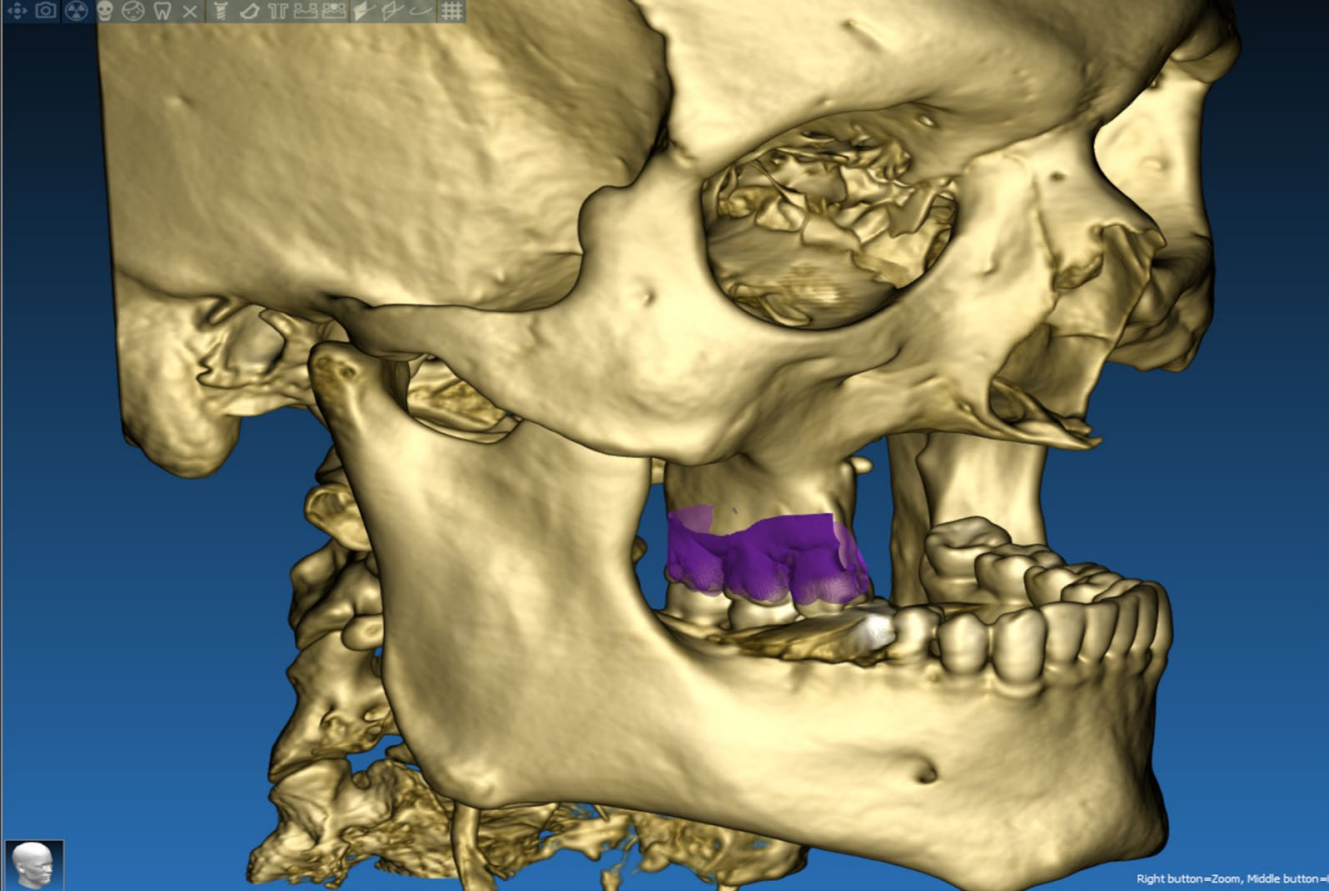
Superimposition between the Dicom file representing the bone and the STL file representing teeth and soft tissue

**Fig. 5:**
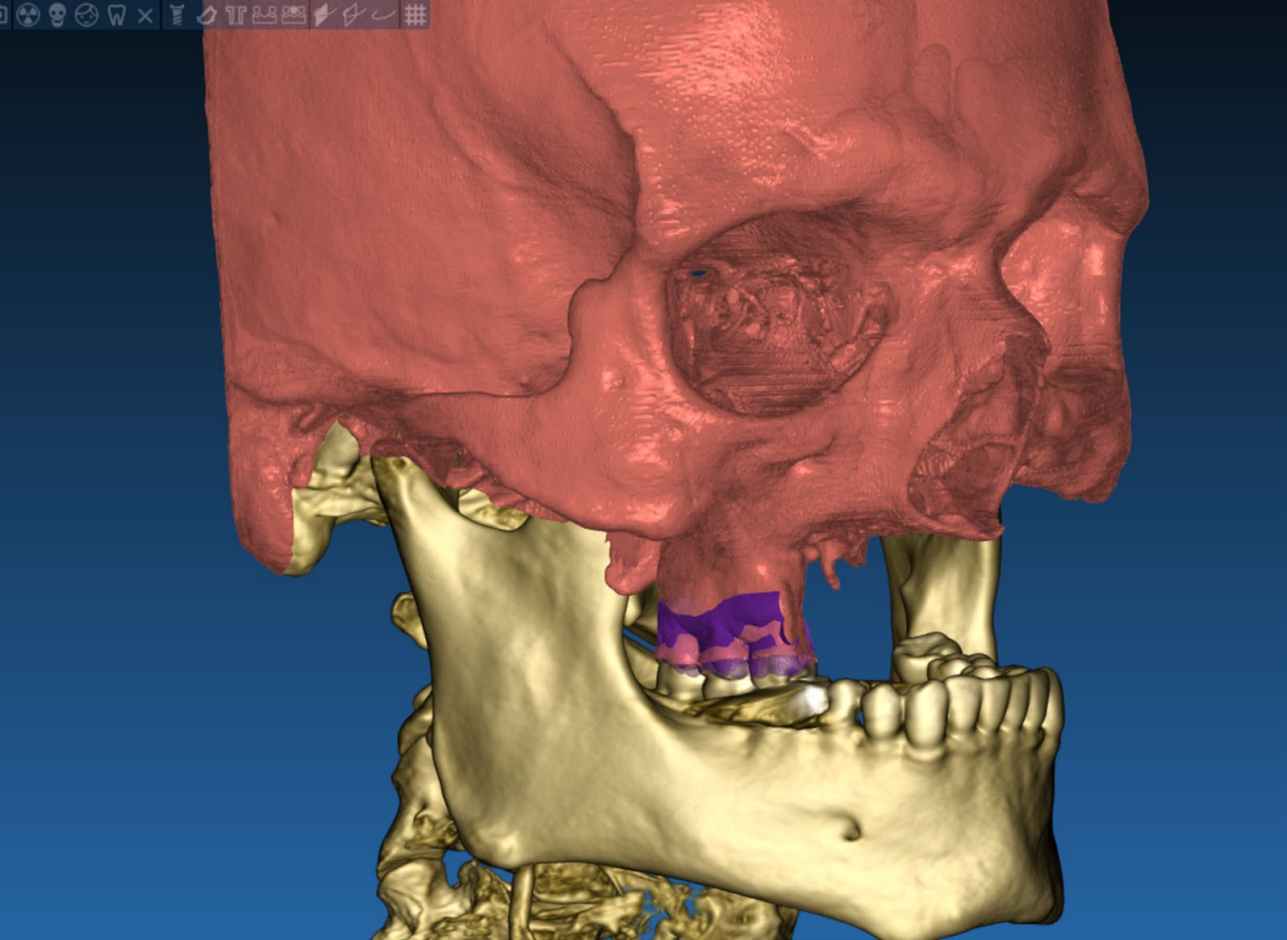
Segmentation of the Dicom file to transform the area of interest into an STL file

**Fig. 6:**
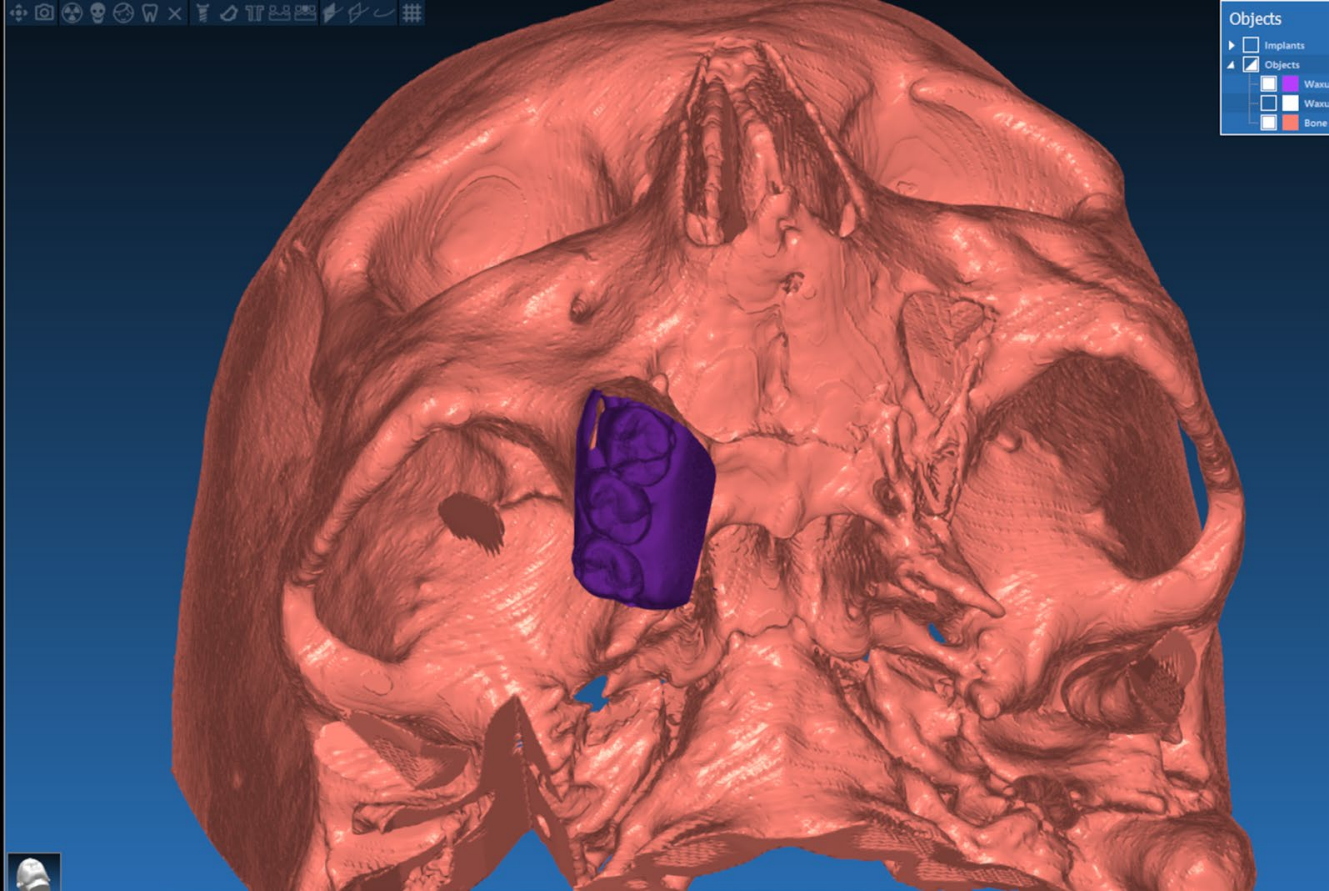
Combining the bone, teeth, and soft tissue data in one STL file

**Fig. 7:**
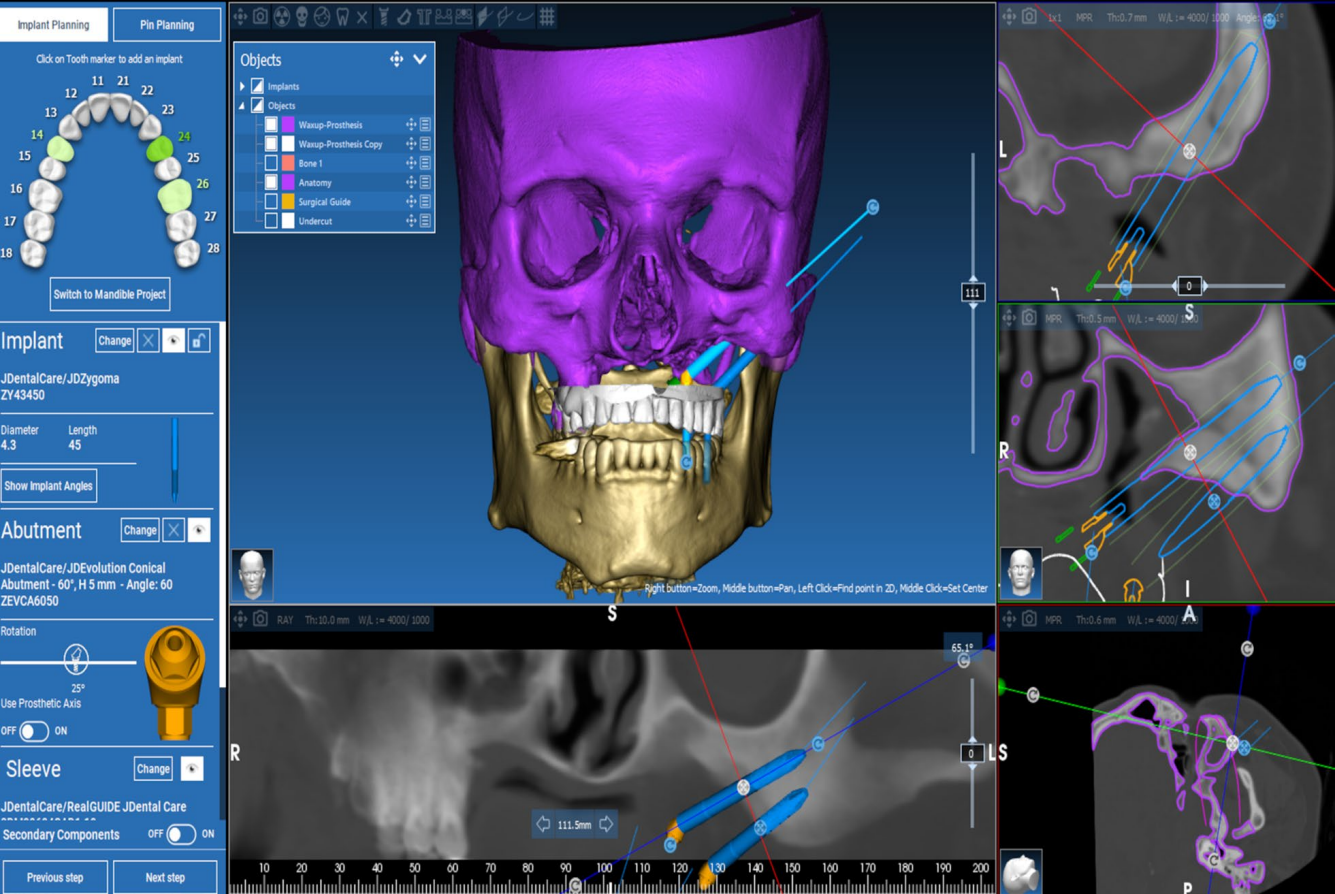
Implant planning according to prosthetically driven implant placement protocol

**Fig. 8:**
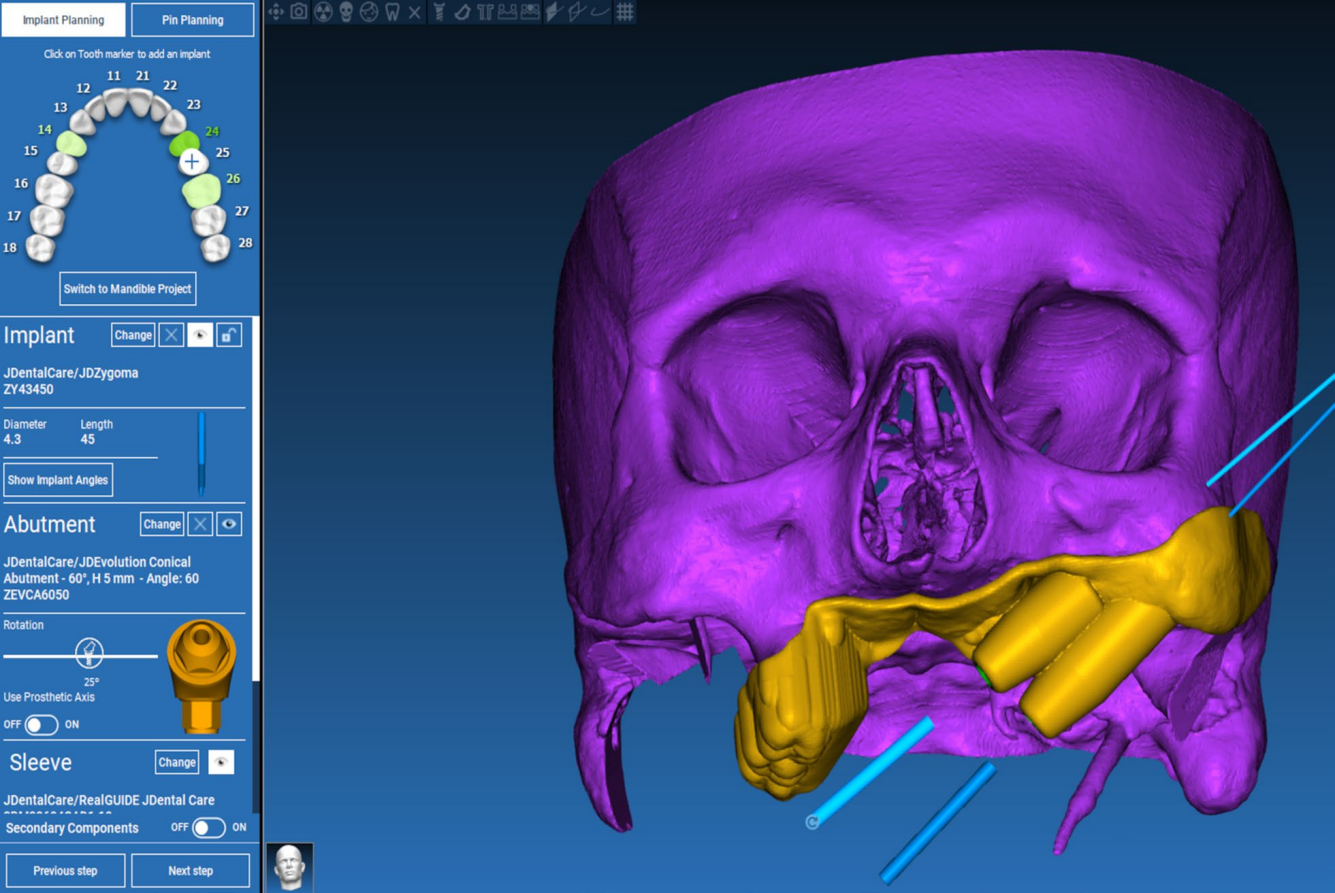
The final design of the hybrid teeth and bone-supported surgical guide

**Fig. 9:**
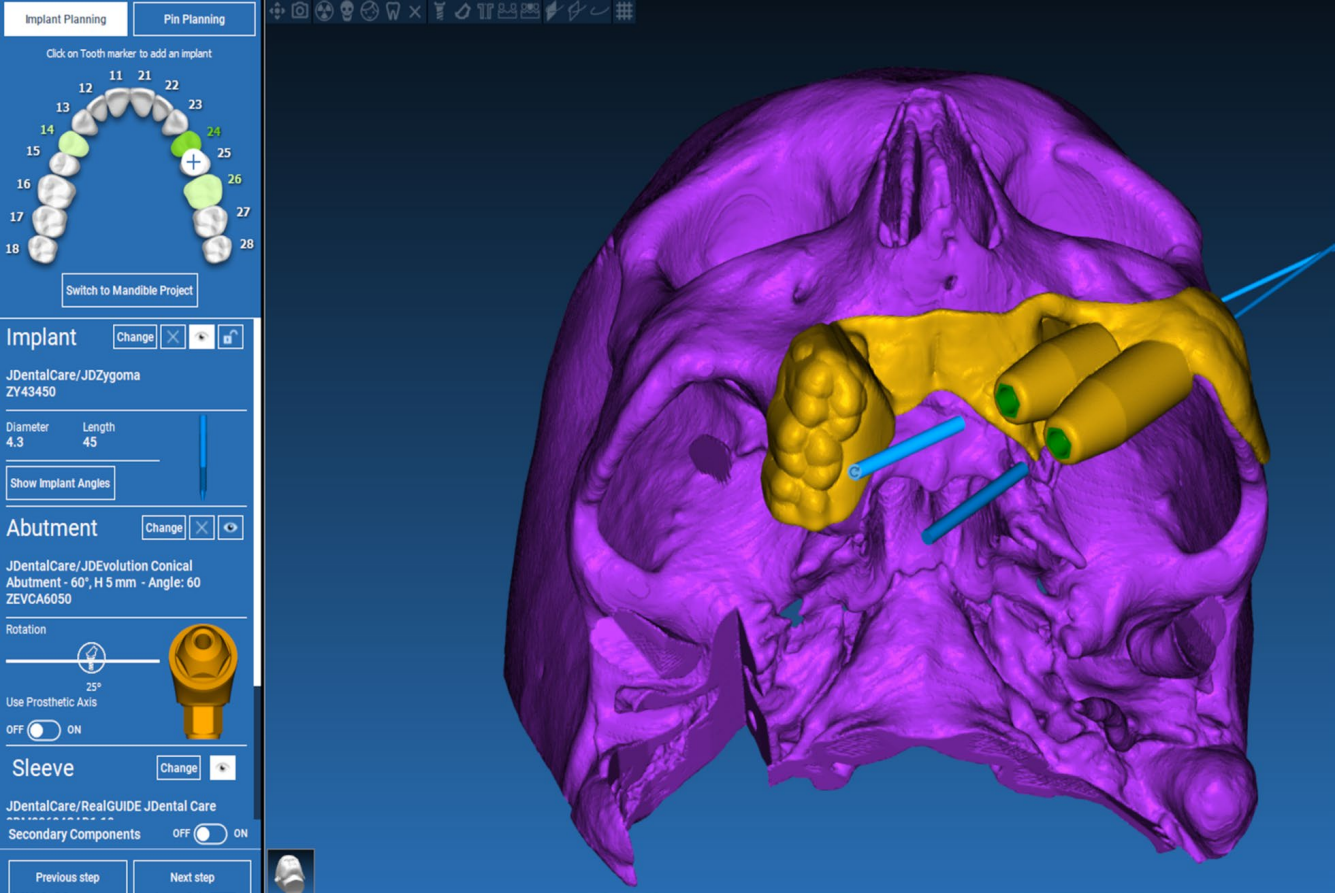
The final design of the hybrid teeth and bone-supported surgical guide

**Fig. 10:**
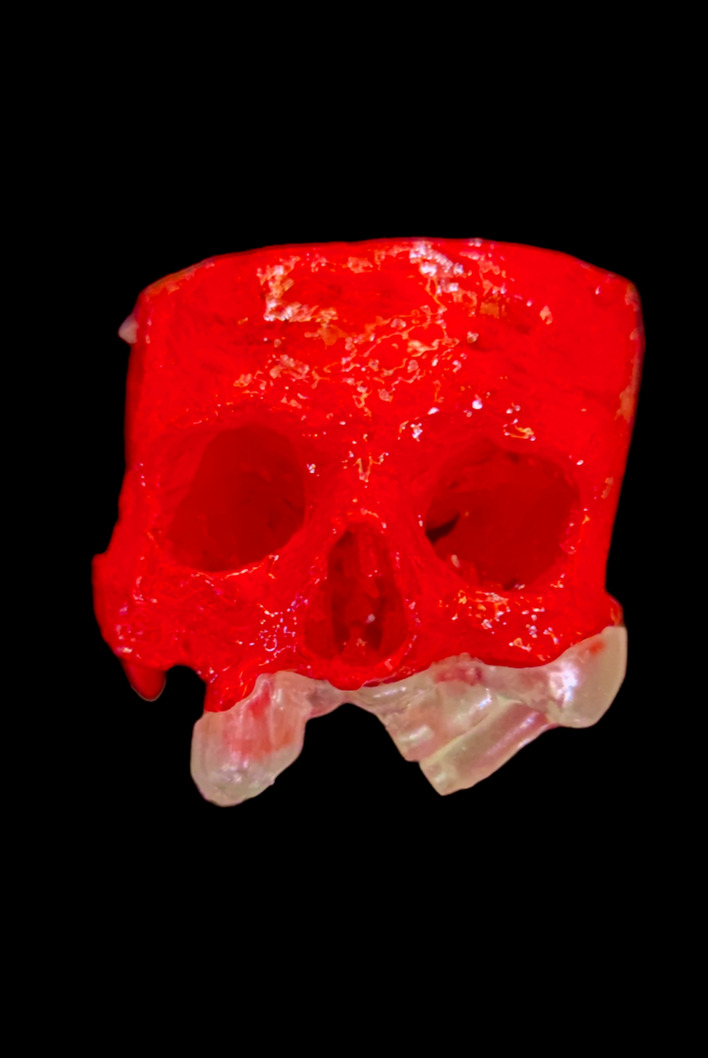
The 3D printed skull (red in color) and the 3D printed surgical guide in clear resin

1. Obtain a CBCT scan of the patient using (PaX-i3D Green, VATECH), and export the data in the form of a Digital Imaging and Communication in Medicine (DICOM) file. The DICOM file represents the bone and teeth surfaces (Fig. 1).
2. Make an optical scan of the remaining natural teeth either directly intraorally or indirectly by extraoral optical scanning of a conventional impression (Medit i700; Medit), then export the data in the form of a standard tessellation language (STL) file. The STL file represents the teeth surfaces and the surrounding soft tissue [13] (Fig. 2).
3. Import both the STL and DICOM files into an implant planning and surgical guide design software program (Real Guide 5.0 software, 3DIEMME).
4. Use the sandbox panel and trim any data other than the teeth and their surrounding soft tissue that will provide support for the future surgical guide (Fig. 3).
5. Align the Dicom and the STL files through the built-in software artificial intelligence using an assisted alignment software tool, or you can also align the two data files by picking up similar points in the two files, followed by best-fit alignment (Fig. 4).
6. Use the segmentation panel to convert the bone Dicom file into an STL file on which a surgical guide can be designed [14]. The segmentation is made by adjusting the bone threshold and using the Select software tool to choose your area of interest only. At the end of this step, you will have two STL files; one representing the bone and the other representing the teeth of interest and their soft tissue surrounding (Fig. 5).
7. Merge the two STL files into one STL file using the sandbox software panel and the Boolean union software tool [15] (Fig. 6).
8. After determining the panoramic curve, start with a virtual setting of the missing teeth and plan the implant position, length, and diameter according to the prosthetically driven implant concept. You can also choose the sleeve diameter and offset according to the drill length if you are using a universal guided surgical kit (Fig. 7).
9. To design the surgical guide, start with selecting the surgical guide bath of insertion by blocking out any unfavorable undercuts, followed by drawing the surgical guide borders, and finally guide generation (Figs. 8, 9, Additional file 1: Video S1). You can control the guide thickness, but it is better not to be less than 3 mm to prevent surgical guide breakage during the surgery. You can also add an oval hole in the surgical guide as a reference for complete guide seating during the surgery.
10. Export the finalized surgical guide design in the form of an STL file and then 3D print the file in clear surgical guide resin (EPAX Resin, EPAX 3D) (Fig. 10).

## Discussion

This is a step-by-step technique to digitally design and fabricate an implant drilling surgical guide that gains its support from both bone and remaining natural teeth, especially valuable in distal extension partially edentulous patients with few remaining natural dentitions. Previous reports have described different types of implant placement surgical guides [3, 16–19]. Still, the authors are unaware of any previous articles on hybrid bone and teeth CAD-CAM fabricated implant drilling surgical guides.

The challenge addressed in this technique is to digitally combine the bone, teeth, and soft tissue data in one STL file using the available CAD technology to fabricate this hybrid surgical guide that can be used in a few remaining natural teeth and long distal extension edentulous areas. This technique introduces both teeth and bone-supported guides, increasing the stability and accuracy of implant placement surgical guides either in the maxilla or mandible with long-span edentulous area [10] or even in patients with hemi maxillectomy requiring either zygomatic or pterygoid implants.

Many studies reported that implants placed using surgical guides in distal extension arch spaces resulted in significantly lower accuracy and higher deviation in implant position in comparison to implants placed in supported sites bilaterally, which could be attributed to the bending tendency of the surgical guide during the drilling sequence to prepare the osteotomy in long span free end saddle edentulous situations [8, 10, 20].

Using the introduced combined bone- and teeth-supported additively manufactured surgical guide, operators can precisely transfer the prosthetically planned implant angulation, and depth from the implant software to the patient's mouth. Which in turn prevents any compromised prosthetic and mechanical implant failures [4]. The bone-supported portion used in this technique provides a vertical stop preventing the guide from bending and in turn placing the implant in an inaccurate position.

The described technique is not routinely used for guided implant placement, as these hybrid surgical guides are used only when there are few unilateral remaining natural teeth. However, conventional surgical guides are recommended to be used with the bilateral presence of natural teeth in relation to the edentulous area.

Limitations of the technique include the need for a CAD designer with high skills in mastering the implant planning software program and the jaw segmentation procedures used in this technique to fabricate bone-supported guides. This type of hybrid surgical guide requires elevating a soft tissue flap for complete guide seating to gain bone support. Clinical and in vitro studies are recommended to evaluate the accuracy of this newly introduced type of implant placement surgical guide and compare its performance with the previously introduced surgical stents, which will reduce potential complications with a more predictable prosthetically driven implant placement.

## Summary

The introduced technique depends on digital technology to design and fabricate a hybrid bone and teeth-supported implant placement surgical guide that could be indicated in many clinical situations where there are few remaining natural teeth in free end saddle partially edentulous patients.

### Supplementary Information

**Additional file 1**: The full digital workflow to design a hybrid bone and tooth supported surgical guide.

## Data Availability

The datasets used and/or analyzed during the current study are available from the corresponding author upon reasonable request.

## Abbreviations

CAD-CAM: Computer aided design-computer aided manufacture
CBCT: Cone beam computed tomography
DICOM: Digital imaging and communication in medicine
STL: Standard tessellation language
